# Assessing the usefulness and acceptability of a low health literacy online decision aid about reproductive choices for younger women with breast cancer: the aLLIAnCE pilot study protocol

**DOI:** 10.1186/s40814-017-0144-9

**Published:** 2017-06-07

**Authors:** Michelle Peate, Sian Karen Smith, Victoria Pye, Alice Hucker, Catharyn Stern, Lesley Stafford, Catherine Oakman, Laura Chin-Lenn, Kerry Shanahan, Nipuni Ratnayake Gamage, Martha Hickey

**Affiliations:** 1 Psychosocial Health and Wellbeing Research (emPoWeR) Unit, Department of Obstetrics & Gynaecology, University of Melbourne, Level 7, Royal Women’ Hospital, 20 Flemington Road, Parkville, VIC 3052 Australia; 2 Psychosocial Research Group, Prince of Wales Clinical School, Faculty of Medicine, UNSW, Lowy Research Centre C25, Level 4, Sydney, NSW 2052 Australia; 3 Australian Institute of Health Innovation, Macquarie University, Sydney, NSW 2109 Australia; 4 Endocrine and Metabolic Service and Reproductive Services, Royal Women’s Hospital and Melbourne IVF, 20 Flemington Road, Parkville, VIC 3052 Australia; 5 Centre for Women’s Mental Health, The Royal Women’s Hospital, 20 Flemington Road, Parkville, VIC 3052 Australia; 6 Breast Service, Royal Melbourne and Royal Women’s Hospitals, Parkville, VIC 3052 Australia; 7 Breast Service, Royal Melbourne Hospital, Parkville, VIC 3052 Australia; 8 Department of Obstetrics & Gynaecology, University of Melbourne, Level 7, Royal Women’s Hospital, 20 Flemington Road, Parkville, VIC 3052 Australia

**Keywords:** Breast cancer, Fertility, Fertility preservation, Decision-making, Young women, Decision aid, Low health literacy, Psychosocial issues, Unmet needs

## Abstract

**Background:**

Young women diagnosed with breast cancer may be confronted by many difficult decisions, especially around fertility preservation prior to commencing cancer treatment. The information to be conveyed is complex, and it may be difficult to weigh up the risks and benefits of the different fertility preservation options available. This complexity is compounded by the widespread low levels of literacy and health literacy in Australia, which may result in greater difficulties in understanding available health information and in decision-making.

**Methods/design:**

A working group of experts have developed a fertility-related online decision aid for a low health literacy population, guided by health literacy principles. The decision aid will be pilot tested with 30 women diagnosed with early breast cancer between 5 years and 6 months previously. To be eligible, at the time of diagnosis, women must be between 18 and 40 years (inclusive), pre-menopausal, have no history of metastatic disease, have not completed their families, be able to give informed consent and have low health literacy. Participants will be asked to reflect back to the time in which they were diagnosed. Participants will complete a questionnaire before and after reviewing the decision aid to determine the feasibility, use and acceptability of the decision aid. The decision aid will be modified accordingly. Participants may also choose to review a previously developed (high literacy) decision aid and provide feedback in comparison to the low health literacy decision aid.

**Discussion:**

This project represents the first study to develop an online fertility decision aid developed from low health literacy models in the context of breast cancer. It is anticipated that the low health literacy decision aid will be useful and acceptable to young women with low health literacy who have been diagnosed with breast cancer and that it will be preferred over the high literacy decision aid.

**Trial registration:**

ACTRN12615001364561p

## Background

Breast cancer is the most frequently diagnosed cancer in reproductive aged women [1, 2]. In Australia, approximately 1000 women annually are under 40 years of age at the time of diagnosis [1]. Prognosis is often excellent, but adjuvant chemotherapy commonly results in permanent ovarian failure, depending on age at the time of treatment and dose and type of chemotherapy used [3]. Furthermore, delaying pregnancy until completion of endocrine therapy will be associated with an age-related reduction in fertility and may result in women being past their reproductive age by completion of treatment. Additionally, as women are starting their families at older ages than in previous decades [4], breast cancer in women who are childless or have not completed their families at diagnosis is becoming more common. For many, the impact of cancer treatment on future fertility is a significant issue [5].

Fortunately, for many young women, there are fertility preservation options available. It is essential that women have access to high-quality information in order to make decisions about these interventions. Previous research has found that many young women are not fully informed [5–8]. Patients who are better informed experience greater emotional, social and physical well-being [9]; better clinical outcomes; better quality of life [9]; and improved satisfaction with care [10]. Thus, good management must include consideration of fertility information needs [11].

To address these needs, a fertility decision aid that improved knowledge and satisfaction while reducing uncertainty and regret has been previously developed [12–14]. However, there is already growing evidence of poor outcomes for people with low health literacy with the use of decision aids that do not target this group [15]. In a systematic review of patient decision aid trials, lower health literacy was associated with lower patient health knowledge, higher decisional uncertainty and regret and lower desire for involvement in the decision-making process and less question-asking and less patient-centred communication [15]. Considering this evidence of poorer outcomes and that 52% of Australian women aged 15 to 44 years do not have the minimum literacy skills required to ‘meet the complex demands of everyday life’ [16], it is highly likely this population will not benefit from the existing decision aid. Specifically, the decision aid does not align with the updated International Patient Decision Aids Standards (IPDAS) recommendations for low health literacy as the language of the existing text-dense decision aid was not written at a level that would be understood by the majority of a low health literacy group, nor was it developed using good health literacy principles [15]. As such, there is an argument that provision of information that is not targeted for low health literacy is inappropriate, especially in the context of making informed decisions [17, 18].

Health literacy is defined as the knowledge and skills required to understand and use information relating to health issues [16]. Those with low health literacy may lack the cognitive and social skills that determine the capacity to gain access to, understand and use information to support good health [19]. They are often disempowered because they receive less information, ask fewer questions and are less satisfied with health care provider communication [20]. Furthermore, low health literacy is associated with a wide range of adverse health outcomes, including reduced overall health status and poorer self-care management [21, 22]. Thus, low health literacy groups, such as culturally and linguistically diverse and indigenous groups, are socially disadvantaged. Additionally, even people who are usually considered to have high literacy may have low health literacy when faced with a difficult diagnosis [23]. It is hoped that targeted information will help those in this low health literacy group to cope with the information overload that often occurs [24]. Few decision aids have been developed for low health literacy populations, and this is a recognised gap in consumer resources [17, 25].

Recently, a Dutch fertility decision aid was developed for women with breast cancer with lower education [26] and found that the improvement in knowledge of less educated women was less than the improvement seen in the better educated women, after receiving the decision aid [27]. Although the Dutch decision aid showed differences in outcomes according to education level, it did not specifically target or investigate low health literacy. Considering that women with high education can also have low health literacy, the Dutch tool does not address the issues beyond level of language as it was not designed according to the IPDAS low health literacy guidelines. It is not merely the level of language that is important but also the reading, listening, analytical and decision-making skills [28, 29] which must be addressed when developing materials to improve patient understanding. Online interventions are a useful medium for this purpose as it allows for alternative formats for information delivery (other than just text) and can be used for interactive learning, such as teach-back methodology, which have been shown to improve satisfaction and have beneficial effects on health behaviours [15]. Thus, there is an urgent need to develop a low health literacy fertility decision aid developed using low health literacy strategies that can be accessed and understood by all patients.

As the discussion of fertility preservation is recommended at the highly stressful time of cancer diagnosis [30, 31], it is important to evaluate the impact of decision aids [32] to ensure that they do not make an already difficult situation worse. Thus, the aim of this pilot study is to develop and assess the acceptability of a low literacy fertility-related decision aid for young women with early breast cancer and low health literacy. The goal is to ensure that the decision aid would be useful at the time of their diagnosis, would improve their knowledge and would not have a negative emotional impact.

## Methods/design

A two-stage process will be used to ensure that the decision aid is acceptable to patients and is an effective decision tool. This paper reports on the protocol for the development and pilot testing of the decision aid.

The decision aid has been designed in accordance with the latest IPDAS [14] to assist patients with low health literacy reach an informed decision about fertility preservation. Although the aim in this study is to establish acceptability in a low health literacy population, we anticipate this will also be useful for all women (and this will be evaluated in the next phase of the study). It includes information concerning the pros and cons of fertility preservation in women with early breast cancer and has evidence-based representation of chances of success. It addresses the complexity, uncertain benefits and potentially large costs (e.g. emotional, financial and physical) in this setting. A summary of treatment procedures is presented with a set of value-clarification exercises to help weigh up the pros and cons of fertility preservation in light of patient values and life situation.

The decision aid is web-based to allow for the use of various communication forms. It is specifically designed for a low health literacy audience and includes illustrations, audio and video components to communicate topics and enhance understanding [33]. Text has been simplified and lay language used. The content is based on the previously developed fertility-related decision aid [12, 34, 35], a literature review and consultation with experts, including health literacy experts, oncologists, breast surgeons, breast care nurses, reproductive health specialists, psychologists, behavioural scientists and consumers. It is guided by basic education and linguistic theory, health literacy and risk communication research, and the conceptual models of low health literacy, to maximise comprehension [29, 36–38].

### Aim

The aim of this study is to assess the usefulness and acceptability of a low health literacy decision aid for fertility preservation amongst young women with early breast cancer. Additionally, a secondary outcome for women is to assess whether the decision aid improves knowledge.

### Sample size

Recommended sample sizes for pilot studies are 10–30 participants [39–42] or 10% of the sample projected for the larger parent study [43, 44]. As our final goal in stage two (described below) is to recruit 270 women for a final sample of 178, the pilot will comprise 30 women.

### Eligibility criteria

Women with a previous diagnosis of early breast cancer will be recruited. To be eligible to participate, women must, at the time of diagnosis:

- Be aged between 18 and 40 years (inclusive)
- Have had a histologically confirmed diagnosis of early-stage breast cancer
- Be pre-menopausal (regular menstrual periods and no vasomotor symptoms)
- Have had no history of metastatic disease
- Have not completed their families

And currently:

(f) Diagnosed between 6 months and 5 years previously; the lower limit of 6 months was chosen to avoid increased burden on women who are currently on active treatment, and the upper limit of 5 years was selected primarily as most young women with breast cancer are advised to avoid pregnancy for at least 2 years and for some up to 5 years (upon completion of endocrine therapy) and these women may still be thinking about having children. This time window also ensures that we would have the participant numbers from our two sites to provide useful pilot data.
(g) Be able to give informed consent.
(h) Be identified as having low health literacy using the Newest Vital Sign (NVS) health literacy instrument as a screening tool (a 5-min food label quiz structured around reading and understanding information) [45].

Women will also need adequate English skills to complete the questionnaires and communicate with the researchers.

### Procedure

**Fig. 1:**
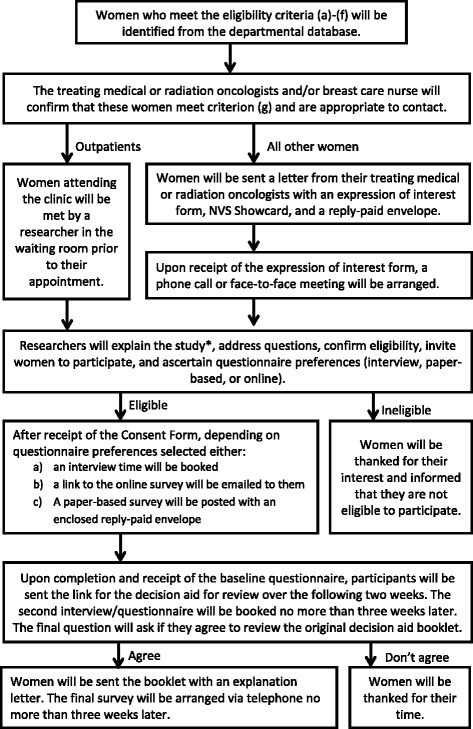
Stage 1 pilot-testing procedure. *The voluntary nature of participation will be explained, including that declining participation or later withdrawal will not impact on medical care

Pilot testing will be conducted through the oncology clinic at a tertiary-level hospital in a major metropolitan centre following the process outlined in Fig. 1. The decision aid will be modified based on the findings from the pilot testing.

### Questionnaires/measures

We will be adapting a questionnaire previously used in related studies to evaluate patient decision support education tools to be suitable for a low health literacy population [34]. Two questionnaires will be administered pre- and post-review of the decision aid. A third, brief, questionnaire will be administered to participants who opt in at the end of the second questionnaire to compare this online decision aid to the original paper-based (high literacy) decision aid [12]. Participants will be given the option to complete the questionnaire online, on a paper questionnaire or via interview. The items in these questionnaires are shown in Table 1.

**Table 1:** Pilot-testing questionnaire content (stage 1)

	Pre	Post	Comparison survey
Demographic data, including current age, age at diagnosis, relationship status, postcode of residence, country of birth, time lived in Australia, Aboriginal or Torres Strait Islander origin, first language spoken, level of English, highest education level, medical and allied health training, employment status, profession, parity and menopausal status.	✔		
Importance of fertility at time of diagnosis (very, somewhat, not at all) and plans for future children (yes/no, when).	✔		
Data on cancer treatment and fertility specialist referrals for previous cancer treatment, as well as fertility treatments accessed.	✔		
Knowledge of breast cancer treatment and fertility preservation assessed using 10 items from a knowledge scale previously used [12], adapted for people with low health literacy. Correct responses will be summed for a knowledge score.	✔	✔	
Acceptability: how the decision aid was accessed (time spent working through materials, thoroughness) and perceptions about the content (amount of information, length, presentation, appeal, ease of reading, order of topics, pace, balance, confusion, direction, clarity).		✔	
Acceptability: how the decision aid was accessed (thoroughness) and perceptions about the content (presentation, appeal, ease of reading, clarity).			✔
Perceived improvement in understanding: six items specifically designed to assess perceived improvement in understanding of the impact of cancer treatment on fertility and of fertility treatment on the cancer prognosis and the pros and cons associated with each available fertility option. Response options ranging from ‘not at all’ to ‘a lot’.		✔	
Satisfaction with the decision aid: evaluating the amount of information, length, balance, direction and presentation of the decision aid using structured response categories. Also, specific questions regarding the ease of understanding of particular sections of the decision aid will be included.		✔	
Emotional impact of the decision aid: if the decision aid (or specific sections of the decision aid) is reassuring, causes worry, concern or distress, and whether the decision aid would have helped them cope better with their situation. Response options ranging from “not at all” to “very much so”.		✔	
Relevance of the decision aid: perceived relevance of the decision aid will be determined using two Likert-style questions asking participants to indicate how relevant they felt the information would have been at the time of diagnosis and how helpful the decision aid would have been in reaching their decision. One item will assess whether participants would recommend the decision aid to others in the same situation.		✔	
Open-ended questions: space will be provided for participants to comment on their satisfaction with the decision aid, the relevance of the decision aid to their situation and the emotional impact of the decision aid. Women will also be asked to identify areas which require more or less detail, their preferred order of topics and for their general suggestions for improvement.		✔	
Comparison of decision aids: selection of which decision aid was more helpful and why; selection of which decision aid was easier to read and understand and why; overall preferred decision aid and why.			✔

### Analysis

Basic descriptive statistics, including means, medians, percentages, ranges and standard deviations, and 95% confidence intervals, will be calculated to describe the sample in terms of socio-demographic characteristics. The comparison survey will be analysed descriptively.

## Discussion

Fertility concerns continue to be an important issue for young women diagnosed with breast cancer. These decisions are often made under pressure of time and may be complex, especially as it is a rapidly changing field. This is compounded by the widespread low levels of literacy in Australia, which may result in greater difficulties in understanding available health information and in decision-making.

Women require decisional support in this area [8]. This has been addressed through the development of interventions such as decision aids [12, 27, 34]; however, these interventions are unlikely to cater for the large proportion of women with low health literacy [16]. The lack of decision support widens the disadvantage gap, with these women unlikely to have their information needs met. Thus, there is a need for tools that are specifically targeted to this population [17].

Additionally, the decision aid may be of benefit to women who are not identified as having low health literacy. Women faced with a breast cancer diagnosis are often feeling overwhelmed and need to make a number of important treatment decisions in a relatively short amount of time, and thus, a decision aid that supports the processing of complex information in a simple manner may be appropriate for all women in this group.

This project will represent the first study to develop a fertility decision aid that supports women with low health literacy to be actively involved in their health care decisions about fertility preservation in the context of breast cancer. The evidence-based tool was developed by experts in this field according to international guidelines for decision aids [14] and using low health literacy models. This pilot study will evaluate the acceptability and perceived usefulness of this decision aid amongst young women with a previous history of breast cancer to provide support for the prospective use of this tool. It is anticipated that not only will this decision aid be acceptable and useful to women but it also will improve knowledge without having negative emotional impact, and they would recommend the decision aid to others in a similar situation. It is also anticipated that participants will report the low health literacy decision aid as more useful and acceptable than the original tool. Future steps will include the prospective evaluation of the low health literacy decision aid in a cluster randomised controlled trial, followed by implementation of the decision aid into clinical practice.

## Abbreviations

aLLIAnCE: **L**ow **L**iteracy Decision **A**id about Reproductive Choices for Younger Women with Breast **C**anc**e**r
IPDAS: International Patient Decision Aid Standards
NVS: Newest Vital Sign health literacy instrument
